# Genetic diversity of the wild ancient tea tree (Camellia taliensis) populations at different altitudes in Qianjiazhai

**DOI:** 10.1371/journal.pone.0283189

**Published:** 2023-04-18

**Authors:** Fei Wang, Xiaomao Cheng, Shoumeng Cheng, Wanting Li, Xiaoxia Huang

**Affiliations:** 1 Ancient Tea Resources Reserve and Utilization Research Center of Yunnan Province, College of Landscape Architecture and Horticulture Sciences, Southwest Forestry University, Kunming, China; 2 Chengdu Institute of Biology, University of Chinese Academy of Sciences, Chengdu, China; ICAR - National Research Center on Plant Biotechnology, INDIA

## Abstract

In this study, the genetic diversity and population structure of 4 wild ancient tea tree (*Camellia taliensis*) populations at different altitudes (2,050, 2,200, 2,350, and 2,500 m) in Qianjiazhai Nature Reserve, Zhenyuan country, Yunnan province, were investigated using EST-SSR molecular markers to compare their genetic variation against altitude. In total, 182 alleles were detected across all loci, ranging from 6 to 25. The top one informative SSR was CsEMS4 with polymorphism information content (PIC) of 0.96. The genetic diversity of this species was high, with 100% of loci being polymorphic, an average Nei’s gene diversity (*H*) of 0.82, and Shannon’s information index (*I*) of 1.99. By contrast, at the population level, the genetic diversity of wild ancient tea tree was relatively low, with values of *H* and *I* being 0.79 and 1.84, respectively. Analysis of molecular variance (AMOVA) revealed a minor genetic differentiation (12.84%) among populations, and most of the genetic variation (87.16%) was detected within populations. Using population structure analysis, we found that the germplasm of wild ancient tea tree was divided into three groups, and there was a substantial gene exchange among these three groups at different altitudes. Divergent habitats caused by altitudes and high gene flow played important roles in genetic diversity of wild ancient tea tree populations, which will provide new opportunities for promoting their protection and potential utilization.

## Introduction

As the basis of life evolution and adaptation, the level of genetic diversity can reflect the adaptability of species with environmental changes. The genetic diversity of plant populations is not influenced only by genotypes, but also by external factors such as geographic areas, altitude, climate, and soil types. The complex environmental aspects (light intensity, temperature, moisture, soil conditions, etc.) at different altitudes have an important influence on the phenological population size; hence, altitude as one of the most critical ecological factors has a strong impact on plant distribution [1]. Previous studies showed that altitude significantly affected the genetic diversity of plants due to different environmental conditions. For example, genetic diversity of plant populations increased or decreased with increasing altitude [2,3], with the genetic diversity of populations being higher at medium compared with low and high altitudes [4]. The genetic diversity of plant populations plays a vital role in their long-term survival, but it may be lost if they become genetically isolated [5]. By contrast, the population longevity and the effective seed and pollen dispersal can increase the resistance to negative effects of the climate and environmental change [6]. Therefore, it is particularly important to clarify the response and adaptation of plants to environmental changes, especially the genetic variation and relationships in different habitat conditions [7].

The wild ancient tea tree is an irreplaceable germplasm resource mainly distributed in the natural forest, and plays an important role in scientific research on the origin, breeding and varietal improvement of tea tree [8]. The wild ancient tea tree has a strong environmental adaptability, containing many genes for the desirable traits such as high contents of catechin, amino acids and polyphenols, and low caffeine, which may be associated with strong resistance of wild ancient tea tree to diseases, pests, drought and cold [9]. However, high heterozygosity combined with a long breeding period complicate traditional breeding of wild ancient tea tree. The abundance of wild ancient trees has been decreasing in recent years due to habitat destruction and other human-induced factors. Hence, there is an urgent need to find a scientific solution to these problems. Yunnan, located in the southwest of China, is the origin of tea trees in the world and has the excellent natural conditions, long history of tea-making and tea-planting [10]. There are abundant germplasm resources of wild ancient tea plants with the crucial scientific and preservation values for studying the origin and enhancing the breeding of tea plants. Although an important progress has been made in understanding the genetic diversity of tea germplasm resources in China, there are few reports on genetic traits of ancient tea plants species in their original habitat [11,12].

Qianjiazhai Nature Reserve is located in the core area of Ailao Mountains, Zhenyuan County, Yunnan Province, China. The wild ancient tea tree (*C*. *taliensis*) community is the dominant plant population in Qianjiazhai Nature Reserve, and is the largest and best-preserved wild ancient tea tree community in the world [13]. In our previous study, we explored the rhizosphere soil, leaf anatomical structure, stoichiometry, and physiological characteristics of ancient wild tea tree populations at different altitudes in Qianjiazhai Nature Reserve [8,14,15]. We found that morphological structure and physiological properties of leaves, the physicochemical characteristics of rhizosphere soil, and the bacterial diversity and community composition in the rhizosphere of ancient wild tea at different altitudes were affected by the habitat heterogeneity. Therefore, the elevational gradient environments of Qianjiazhai Nature Reserve can be used as an ideal study area to explain how various environmental factors influence the genetic characteristics of the wild ancient tea tree communities. Furthermore, it is necessary to assess the genetic diversity of wild ancient tea tree, which can provide some guidelines for subsequent management and protection, given the importance of the species in this region.

Molecular markers are frequently used to analyze the effects of external factors on genetic structure of plant populations [16]. In particular, the third-generation molecular marker EST-SSR (Expressed Sequences Tags-Simple Sequence Repeats) has a potential in a wide range of studies on gene tagging in phylogenetically related species, such as *Monochasma savatieri*, cultivated tea in Sri Lanka and *Cinnamomum camphora* [17–19]. These markers are highly efficient in detecting genetic diversity due to their high polymorphism. In the present study, the genetic diversity and population structure of the wild ancient tea tree (*C*. *taliensis*) populations at different altitudes were determined by the EST-SSR molecular markers. Our purpose was to analyze the population-level genetic variation of the wild ancient tea tree resources of Qianjiazhai Nature Reserve in Yunnan Province. The results may contribute to the theoretical basis for the genetic resource conservation, breeding and innovative utilization of superior varieties of wild ancient tea plants in the future.

## Materials and methods

### Study sites and sampling

Qianjiazhai Nature Reserve (24°17’N, 101°14’E) is located in Zhenyuan County, Yunnan Province, southwest of China. In this region, the average annual temperature is 10~12°C, precipitation is above 1,500 mm, and rainy season is mainly in July and August. The seasonal temperature difference is small, and the frost-free period is from April to October. The Reserve features vertical zonal vegetation of southern subtropical evergreen forest, positioned at the northern margin of the mid-subtropical zone. Wild ancient tea trees (*C*. *taliensis*) are mostly about 1,000 years old and distributed mainly at the altitude of 2,000 to 2,500 m. And the rising altitudes can be a contributing factor to climate and habitat changes in this region [20]. Especially at the altitudes with vertical distances above 100 m, the environmental heterogeneity has an important impact on the growth, morphology, physiological metabolism of trees [21,22]. In this study, the leaf samples of wild ancient tea trees grown at different altitudes (Pop1: 2,050 m, Pop2: 2,200 m, Pop3: 2,350 m, and Pop4: 2,500 m) were collected and stored in silica gel. Between 20 and 26 individual adult trees were sampled in each population. A total of 86 individuals of wild ancient tea trees (*C*. *taliensis*) were sampled from four altitudes as a whole to analyze genetic diversity at the population level; moreover, they were split into considered as four populations (based on altitude) for evaluating the genetic diversity at the species level (Table 1). The sampled trees were at least 50 meters apart to minimize a chance of collecting the genetically-related materials.

**Table 1:** General situation of land samples at different altitudes.

Population code	Altitude(m)	Sample size	Geographic coordinates	Sample type	General situation of habitat
**Pop1**	2050	26	24° 27′ 84″ N, 101° 26′ 37″ E	Leaf	interference, more plants from the families Theaceae, Magnoliaceae, Fagaceae, and Rosaceae without insect pests, shade under the forest
**Pop2**	2200	20	24° 27′ 49″ N, 101°27′ 24″ E	Leaf	Less human interference, fewer Theaceae and Magnoliaceae species suffering from lower insect pressure, unoccupied land under the forest
**Pop3**	2350	20	24° 28′ 93″ N, 101° 26′ 12″ E	Leaf	Without human interference, plenty of ground cover and ferns in forest suffering strongly from insect pests
**Pop4**	2500	20	24° 29′ 48″ N, 101° 26′ 25″ E	Leaf	Without human interference, few ferns with a low insect pressure

Pop1: 2050 m, Pop2: 2200 m, Pop3: 2350 m, Pop4: 2500 m.

### DNA extraction and EST-SSR analysis

The improved CTAB method was used to extract genomic DNA from sampled leaves in this study [23]. We used a Beckman spectrophotometer (DU800, USA) to evaluate the quality and quantity of DNA, ultimately adjusting to an appropriate concentration of 25 ng·uL^-1^ in TE buffer (10 mM Tris, 1 mM EDTA, pH 8.0). Among the 207 pairs of primers that were independently developed by our own lab, sixteen polymorphic EST-SSR markers were selected (S1 Table), and FAM (blue) or HEX (green) fluorescent dye was added to the forward primer synthesized by Sangon Biotech Ltd. Co. (Shanghai). PCR amplifications were performed in a volume of 10 uL including 100 ng of genomic DNA, 1x Taq buffer (Mg^+^), 0.2 uM of dNTPs, 0.8 of uM each primer, and 0.5 U of Taq DNA polymerase (Fermentas) [24]. The reaction mixture was initially denatured at 94°C for 2 min, followed by 35 cycles of amplification at 94°C for 15 s, 58°C for 30 s and 72°C for 35 s, and a final extension at 72°C for 5 min in a Gene Amp PCR system 9700 (Applied Biosystems). Original data were obtained by performing capillary electrophoresis of amplified PCR products using an ABI 3730XL Analyzer. PCR amplification results of primers are shown in S1 Fig.

### Data analysis

The original data obtained by fluorescence detection were converted into the fragment lengths using Quantity One software. Polymorphic fragments were scored qualitatively as either present (1) or absent (0) in each population; then, a binary data matrix was put into Microsoft Excel, and the results were converted to genotypic data using Data Trans 1.0 [25]. A range of genetic parameters within and among populations were calculated using POPGEN-Eversion 1.3.1 software [26], including Observed number of alleles (*Na*), Effective number of alleles (*Ne*), Shannon’s information index (*I*), Nei’s gene diversity (*H*), Observed heterozygosity (*Ho*), Expected heterozygosity (*He*), Percentage of polymorphic loci (*PPB*), Inbred coefficient inside population (*F*_*is*_), Inbred coefficient of the total population (*F*_*it*_), Colony coefficient of differentiation (*F*_*st*_), and Gene flow (*N*_*m*_). Polymorphic information content (*PIC*) was calculated using Powermarker [27]. We also calculated the Müller index of diversity (*Mu*) by the formula [*Mu* = (n·*H*)/(n-1] [28], because *Mu* index could be considered as the correction of Nei’s gene diversity for the small number of samples. The analysis of molecular variance (AMOVA) was used to analyze the genetic variation within and between the wild ancient tea population*s*. The variance components were tested statistically by nonparametric randomization tests using 1,000 permutations [29]. Discriminant Analysis of Principal Components (DAPC) was conducted by using the *adegenet* package for R 4.0.5 software, and Structure 2.3.4 software was used to analyze the genetic structure of plant populations [30,31].

## Results

### EST-SSR loci diversity

As can be seen from Table 2, sixteen primers screened producing clear bands were used to analyze the 86 adult individuals in the four wild ancient tea tree populations from different altitudes. The182 alleles in total were scored at the EST-SSR loci. The observed number of alleles (*Na*) ranged from 6 to 25, and the average value of *Na* and the Effective number of alleles (*Ne*) per loci were 11.38 and 6.66, respectively. Shannon’s Information index (*I*) varied from 1.31 to 2.90, with a mean of 1.99. Nei’gene diversity (*H*) varied from 0.67 to 0.93 with a mean of 0.82, whereas Müller index of diversity (*Mu*) varied from 0.68 to 0.94 with a mean of 0.83. Observed heterozygosity (*Ho*) and Expected heterozygosity (*He*) ranged from 0.28 to 0.98 (average 0.79) and 0.67 to 0.94 (average 0.82), respectively. The polymorphic information content (*PIC*) values of 16 pairs of primers were all greater than 0.5 and ranged from 0.63 to 0.96, indicating the 16 loci were highly polymorphic.

**Table 2:** Genetic diversity parameters of wild ancient tea tree populations based on EST-SSR markers.

Primer	*Na*	*Ne*	*I*	*H*	*Mu*	*Ho*	*He*	*PIC*
**CsEMS1**	12.00	6.04	2.05	0.83	0.84	0.78	0.84	0.89
**CsEMS4**	25.00	14.85	2.90	0.93	0.94	0.93	0.94	0.96
**CsEMS53**	10.00	8.04	2.19	0.88	0.89	0.72	0.88	0.95
**CsEMS68**	6.00	2.99	1.31	0.67	0.68	0.48	0.67	0.77
**CsEMS71**	13.00	8.80	2.33	0.89	0.90	0.92	0.89	0.95
**CsEMS72**	9.00	3.53	1.60	0.72	0.73	0.95	0.72	0.80
**CsEMS78**	8.00	3.08	1.37	0.68	0.69	0.95	0.68	0.63
**CsEMS141**	14.00	7.97	2.29	0.87	0.88	0.98	0.88	0.93
**CsEMS143**	10.00	5.53	1.88	0.82	0.83	0.81	0.82	0.89
**CsEMS146**	7.00	3.91	1.55	0.74	0.75	0.88	0.75	0.77
**CsEMS155**	8.00	5.98	1.91	0.83	0.84	0.28	0.84	0.88
**CsEMS159**	9.00	4.84	1.82	0.79	0.80	0.67	0.80	0.89
**CsEMS183**	7.00	4.39	1.60	0.77	0.78	0.42	0.78	0.83
**CsEMS189**	9.00	6.79	2.04	0.85	0.86	0.86	0.86	0.93
**CsEMS194**	18.00	10.12	2.54	0.90	0.91	0.98	0.91	0.88
**CsEMS201**	17.00	9.74	2.46	0.90	0.91	0.95	0.90	0.91
**Average**	11.38	6.66	1.99	0.82	0.83	0.79	0.82	0.87

*Na*: Observed number of alleles; *Ne*: Effective number of alleles; *I*: Shannon’s information index; *H*: Nei’gene diversity; *Mu*: Müller index of diversity; *Ho*: Observed heterozygosity; *He*: Expected heterozygosity; *PIC*: Polymorphic information content.

### Genetic diversity

The wild ancient tea tree has higher genetic diversity at the species level, with 100% of loci being polymorphic and with an average Nei’s gene diversity (*H*) of 0.82 Müller index of diversity (*Mu*) of 0.83 and Shannon’s information index (*I*) of 1.99 (Table 3). At the population level, the Nei’s gene diversity (*H*) values varied from 0.78 to 0.80, with average of 0.79, whereas the Müller index of diversity (*Mu*) values varied from 0.82 to 0.84 with average of 0.83. And the values of Shannon’s index (*I*) varied from 1.78 to 1.88, with average of 1.84. Both *H* and *I* values indicated that genetic diversity of wild ancient tea tree at the population level was lower than that at the species level. In addition, as indicated by the Nei’s gene diversity and Shannon’s information index, the variation trend of the genetic diversity of four wild ancient tea tree natural populations at different altitudes was generally consistent.

**Table 3:** Genetic diversity parameters of four natural populations of wild ancient tea tree at different altitudes.

Population	Sample size	Altitude (m)	*Na*	*Ne*	*I*	*H*	*Mu*	*Ho*	*He*	*PPB*
**Pop1**	26	2050	9.56	5.81	1.88	0.80	0.83	0.75	0.82	100%
**Pop2**	20	2200	8.75	5.76	1.84	0.79	0.83	0.85	0.81	100%
**Pop3**	20	2350	8.75	6.02	1.86	0.80	0.84	0.77	0.82	100%
**Pop4**	20	2500	8.31	5.44	1.78	0.78	0.82	0.77	0.80	100%
**Population level**	21.5	-	8.84	5.76	1.84	0.79	0.83	0.79	0.81	100%
**Species level**	86	-	11.38	6.66	1.99	0.82	0.83	0.79	0.82	100%

Pop1: 2050 m, Pop2: 2200 m, Pop3: 2350 m, Pop4: 2500 m. *Na*: Observed number of alleles; *Ne*: Effective number of alleles; *I*: Shannon’s information index; *H*: Nei’ gene diversity; *Mu*: Müller index of diversity; *Ho*: Observed heterozygosity; *He*: Expected heterozygosity; *PPB*: Percentage of polymorphic loci.

### Genetic differentiation

As shown in Table 4, Inbred coefficient within population (*F*_*is*_ = 0.01), Inbred coefficient of the total population (*F*_*it*_ = 0.04) and Colony coefficient of differentiation (*F*_*st*_ = 0.03) all indicated a low level of genetic differentiation among populations of wild ancient tea tree, but with high differentiation within populations. In addition, the AMOVA results also revealed that most of the genetic variation (87.16%) existed within the populations and only 12.84% among the populations. The value of gene flow (*N*_*m*_) among the wild ancient tea tree populations was 8.52, implying that the gene flow of wild ancient tea tree populations was strong.

**Table 4:** Genetic differentiation of tea populations along the altitude gradient.

POPGEN					AMOVA					
**Sample size**	*F*_*is*_	*F*_*it*_	*F*_*st*_	*N*_m_	Source of Variation	**df**	**SSD**	**MSD**	**Variance components**	**Total variance**
86	0.01	0.04	0.03	8.52	Among Populations	3	340.00	113.33	4.02	12.84%
					Within Populations	82	2238.43	27.30	27.30	87.16%

*F*_*is*_: Inbred coefficient within population; *F*_*it*_: Inbred coefficient of the total population; *F*_*st*_: Colony coefficient of differentiation; *N*_*m*_: Gene flow. df: Degrees of freedom, SSD: Sum of squares; MSD: Mean squared deviation.

### Genetic distance and structure

**Fig 1:**
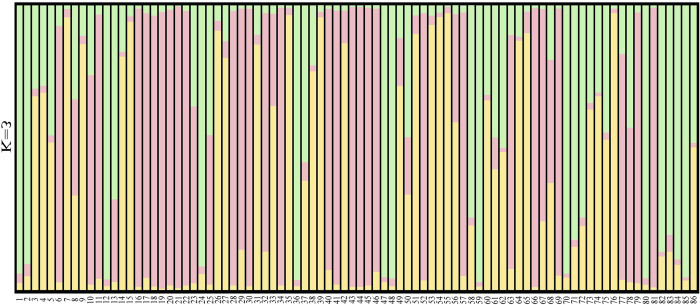
Genetic structure of wild tea tree population at different altitudes in Qianjiazhai Nature Reserve. Different colors indicate three clusters (optimal K = 3). Light green bar represents the first cluster, yellow bar represents the second cluster, pink bar represents the third cluster.

**Fig 2:**
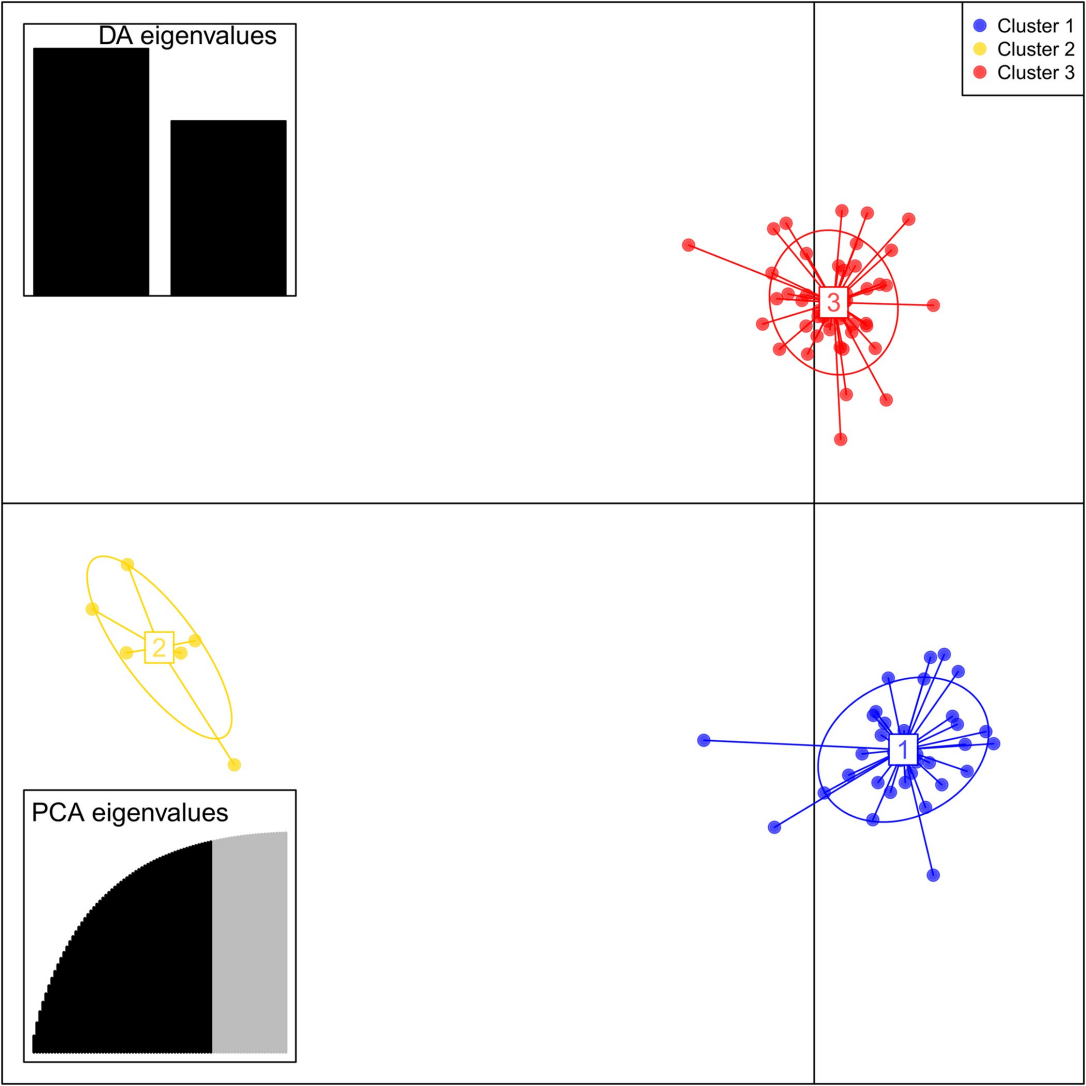
The discriminant analysis of principal components (DAPC) of different wild ancient tea tree populations. The obtained graph represents the individuals as dots and the groups as inertia ellipses. Eigenvalues of the analysis are displayed in inset.

The genetic identity and genetic distance among the four wild ancient tea tree populations were analyzed using POPGENE software (Tables 5 and 6). Nei’s genetic identity (0.88) between Pop1 and Pop2 was the highest, whereas that between Pop2 and Pop4 was the lowest (0.82). The genetic distance was the closest between Pop1 and Pop2 (0.13), and the farthest between Pop2 and Pop4 (0.20). The genetic distance was correlated with altitude, and the genetic distance among the populations at specific elevations was relatively small. A larger difference in altitude meant increased geographical distance of populations, which affected the gene transfer between populations and resulted in larger genetic distance. Based on a genetic structure analysis (Fig 1), we found that the germplasm of 86 wild ancient tea trees in Qianjiazhai Nature Reserve of China was clustered into three groups (K = 3). The classification was not consistent with that based on altitude, and there were frequent gene exchanges among the three clusters. These results on the population structure were also supported by DAPC analysis (Fig 2), with three genetic components detected across four populations.

**Table 5:** Nei’s genetic distance among populations of wild ancient tea tree at different altitudes.

Population	Pop1	Pop2	Pop3	Pop4
**Pop1**	**	-	-	-
**Pop2**	0.13	**	-	-
**Pop3**	0.15	0.18	**	-
**Pop4**	0.16	0.20	0.15	**

Pop1: 2050 m, Pop2: 2200 m, Pop3: 2350 m, Pop4: 2500 m.

**Table 6:** Nei’s genetic identity among populations of wild ancient tea tree at different altitudes.

Population	Pop1	Pop2	Pop3	Pop4
**Pop1**	**	0.88	0.86	0.85
**Pop2**	-	**	0.83	0.82
**Pop3**		-	**	0.86
**Pop4**	-	-	-	**

Pop1: 2050 m, Pop2: 2200 m, Pop3: 2350 m, Pop4: 2500 m.

## Discussion

### Effects of altitudes on genetic diversity

The EST-SSR marker technology was used in this study to reveal the genetic relationships among the wild ancient tea tree populations at four altitudes in Qianjiazhai Nature Reserve of China. The *PIC* values of 16 pairs of primers varied from 0.63 to 0.96, with a mean of 0.87, indicating these primers selected in this study were highly polymorphic [32]. These 16 EST-SSR primers detected the expected heterozygosity (*He*) above 0.5 (Table 2), suggesting that there was a high level of polymorphism among the wild ancient tea tree populations [33]. Compared with previous studies on wild ancient tea tree [12,34], more primers were selected in this experiment and these primers showed high polymorphism in the samples and had strong identification capacity. At the species level, *PPB* was up to 100% which was significantly higher than that of tea plants in other studies, such as the Yunnan tea [*C*. *sinensis* (L.) O. Kuntze] germplasm belonging to 8 species (*PPB* = 98.90%) [35], tea cultivars from China, Japan and Kenya including *C*. *sinensis*, *C*. *sinensis* var. assamica and *C*. *sinensis* var. pubilimba (*PPB* = 99.7%) [36], and *C*. *sinensis* L. (cultivated tea) and its wild relatives (21.4–50.0%) [37]. The *H* was 0.82, *I* was 1.99, and the values of *Ho* (0.79) and *He* (0.82) of 86 tea germplasm resources were similar (Table 3), indicating that genetic diversity of the selected wild ancient tea trees was high. The *He* (0.82) of wild ancient tea tree detected in this research was higher than the SSR-based estimates for other tea species in the same family, such as *C*. *reticulata* (*He* = 0.46), *C*. *saluenensis* (*He* = 0.34), *C*. *pitardii* (*He* = 0.24), and *C*. *yunnanensis* (*He* = 0.17) [38]. These results indicated high genetic diversity of wild ancient tea tree in Qianjiazhai Nature Reserve. This finding is likely to be associated with the unique biological characteristics and living habitats in the Reserve. Firstly, the widespread occurrence of wind pollination promotes outcrossing, which would favor increased genetic diversity of wild ancient tea tree populations [22]. Secondly, many tea trees in this area are more than 1000 years old as the largest and best-preserved wild ancient tea tree community found in the world, so the genes of wild ancient tea tree have been accumulated and preserved in the long-term natural selection, proving that Yunnan province is one of the places of origin of tea tree in the world [15]. The protection of wild ancient tea tree populations in the nature reserve with minimal human interference would also promote high genetic diversity.

At the population level, the values of Nei’s gene diversity (*H*) and Shannon’s information index (*I*) for the four populations of wild ancient tea tree were 0.79 and 1.84, respectively (Table 3). Hence, the genetic diversity was lower at the population than at the species level. Similar findings were reported in *Pteroceltis tatarinowii* [39]. The present study revealed that the genetic diversity estimated by value of *H* was lower than that calculated by value of *I* among the four populations of wild ancient tea tree. The highest genetic diversity was found in the lowest altitude population (2050 m). This indicated an absence of a clear effect of the altitude in this region on the distribution of genetic diversity of wild ancient tea tree populations, which is similar to the research results on *Abies lasiocarpa* [40] and *Nothofagus pumilio* [41]. Such findings may be due to specific combinations of the environmental factors such as water, temperature, light, humidity, and soil around wild ancient tea tree populations changing differently at various altitudes, resulting in differences in the selection pressure exerted on various populations [42]. The population at low altitude is less susceptible to the interference from human activities because of the positive influence of natural reserve [43], contributing to increased species genetic diversity. Moreover, some studies showed that geographical distance among populations (along with altitude) also contributes to genetic differentiation [44,45]. However, in the present study the geographical distance was not a significant factor due to a narrow range of altitudes (2050–2500 m) in which the wild ancient tea tree populations were distributed.

### Effects of altitudes on genetic differentiation

Genetic diversity can indicate the level and distribution pattern of genetic variation, with the genetic differentiation of plant populations affected mainly by their own genetic characteristics and changes in the external environment [46,47]. The AMOVA results showed high variation (87.16%) within populations, and only 12.84% of total variation among the population (Table 4). The value of Colony coefficient of differentiation (*F*_*st*_) was 0.03, which also confirmed that the genetic variation existed mainly within, rather than among, the populations of wild ancient tea tree [48]. A similar variation pattern within populations was observed for *Cymbidium tortisepalum* (89.25%) [49] and *Lilium cernuum* (84%) [50]. This can be explained by the species breeding system, which strongly influences distribution and magnitude of the genetic diversity in plant populations. The seed-setting rate of wild ancient tea tree in natural environment is low, resulting in low genetic differentiation among populations of this species [51]. In general, the perennial, outcrossing plant species retain most of their genetic variability within the population in contrast to annual and self-crossing species [52].

The *N*_*m*_ is considered to indicate genetic differentiation and gene flow among and within populations. If *N*_*m*_ is <1, the dominant factor affecting the genetic structure of population is genetic drift; if *N*_*m*_ is >1, there is sufficient genetic exchanges to prevent the genetic differentiation made by genetic drift among populations [53]. Previous studies [54,55] reported that the *N*_*m*_ values of ancient Chengbudong tea trees population in Xishuangbanna were 1.62 and 7.01, which indicated there was a strong gene flow among the tea plants. So, high gene flow in this study (*N*_*m*_ = 8.52) is a plausible reason for the low genetic differentiation among the wild ancient tea tree populations. This is probably because wild ancient tea tree is one of typical wind-pollinated plants whose pollen can be spread over a long distance. Compared with other tree species, the wild ancient tea tree has some unique biological characteristics, including monoecious, high outcrossing proportion, high tree ages, and over-lapping generations, which contribute to gene migration among the wild ancient tea tree populations and increase heterogeneity among them at different altitudes, thereby reducing the genetic differentiation among populations.

### Effects of altitudes on genetic distance and structure

Genetic distance is one of the common indicators of genetic differentiation among plant populations [56]. In the present study, we found that the genetic distance between the wild ancient tea tree populations at adjacent altitudes was small, and genetic distance over large altitude difference was larger. The effect of altitude difference on the genetic distance of wild ancient tea tree populations indicated that altitude affected genetic differentiation of wild ancient tea tree and hindered gene exchange. Smaller genetic distance of populations at the adjacent altitudes may be due to the similar habitat types, so the natural selection pressure tends to be the same. The results indicated that the genetic distances among populations were correlated with their altitudinal distribution, with the genetic distance being relatively small between the populations at adjacent altitudes. The study site is in the nature reserve where human interference is low, and the small altitude difference minimizes the genetic distance among the low-altitude populations. By contrast, the harsh habitat conditions at high altitudes may affect the survival and reproduction or the genetic flow of the populations, which leads to larger genetic distances [57,58].

We found that 86 wild ancient tea tree specimens in Qianjiazhai Nature Reserve can be divided into three clusters (k = 3) based on the genetic structure analysis (Fig 1), but the numbers of taxa and populations were different, indicating the partly mixed genetic structure of individuals among the four wild ancient tea tree populations at different elevations. Three genetic components were detected in the four populations also by DAPC analysis (Fig 2), supporting the population structure analysis. Importantly the classification at the genetic level was not completely consistent with the altitude from which the genetic material was sourced, with some materials from the same altitude dispersed in other clusters, which might be due to some genes infiltrating into populations at different altitudes by exchange. This is consistent with the results on tea resources in the southwest China obtained by Yao et al [59].

## Conclusions

We studied the genetic diversity of wild ancient tea tree collected from different altitudes in Qianjiazhai Nature Reserve by using EST-SSR molecular markers. The results indicated the high total genetic diversity of wild ancient tea tree populations in Qianjiazhai Nature Reserve because of the area’s unique biological characteristics and living habits. The strong gene flow may be a dominant factor resulting in the high genetic diversity among the wild ancient tea tree populations. Furthermore, altitude affected the genetic diversity of wild ancient tea tree populations in this region. The population of wild ancient tea tree at low altitude (2,050 m) showed a higher level of genetic diversity than the populations at middle and high altitude (2,200 m, 2,350 m, and 2,500 m) probably because environmental disturbance posed a negative effect on the diversity along the altitude gradient, whereas natural reserve played a significant positive role in protecting species diversity. These findings enhance our understanding of the interactions between genetic diversity of species and environmental factors and provide new insights into the factors influencing genetic characteristics of wild ancient tea tree at different altitudes.

## Supporting information

S1 Fig Gel photographs of partial primers amplification products for 86 individual samples of wild ancient tea tree from different altitudes based on EST-SSR marker. A: CsEMS72, B: CsEMS78, C: CsEMS189, D: CsEMS201. Pop1: 1–26, Pop2: 27–46, Pop3: 47–66, Pop4: 67–86.
(TIF)

S1 Table 16 pairs of EST-SSR primers correlation properties. (PDF)
