# Supplementary figures and images for: Genetic diversity of the wild ancient tea tree (Camellia taliensis) populations at different altitudes in Qianjiazhai
Source: PLoS One. 2023 Apr 18;18(4):e0283189. doi: 10.1371/journal.pone.0283189 (PMC10112783; doi:10.1371/journal.pone.0283189)

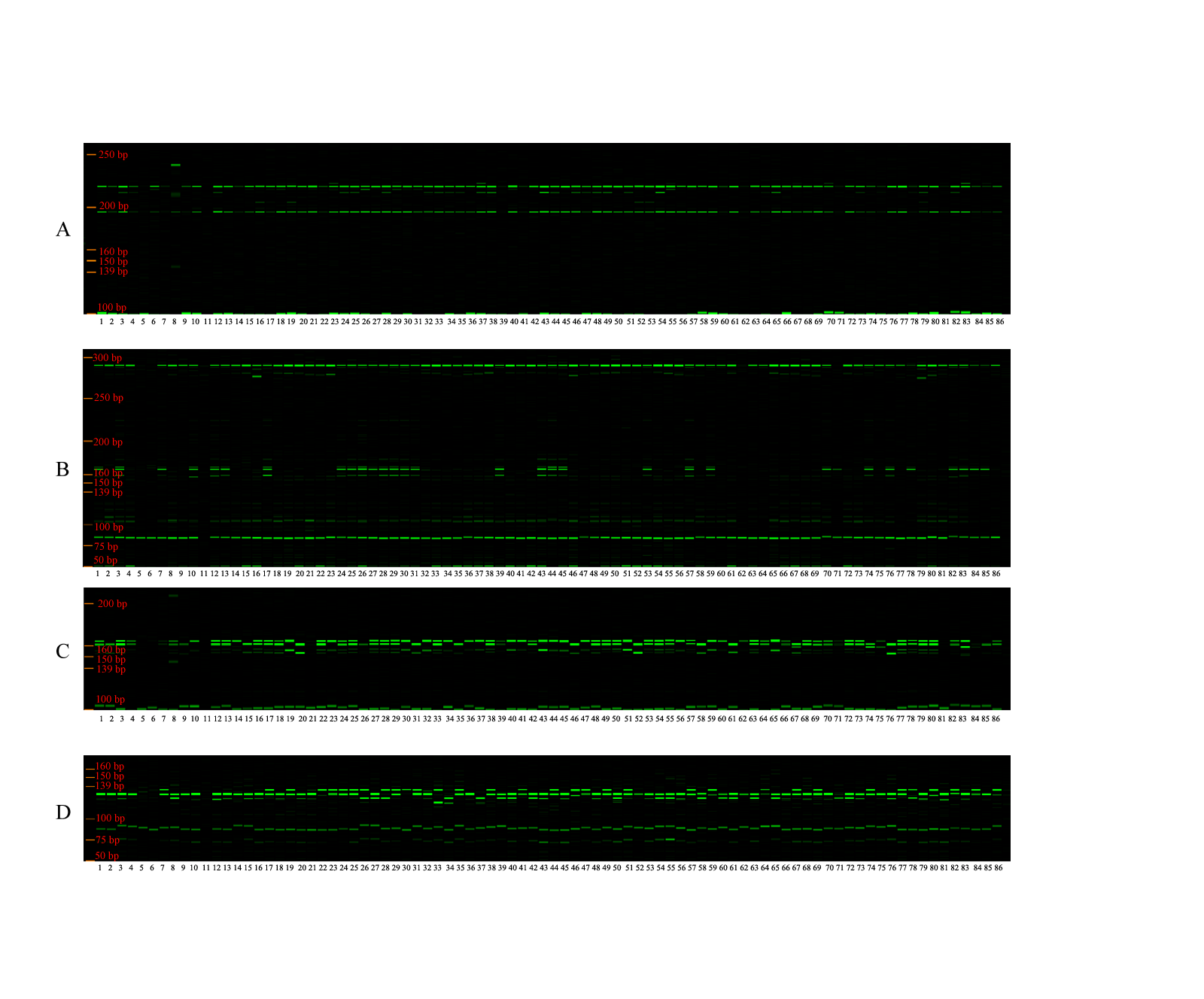

Supplement: S1 Fig — A: CsEMS72, B: CsEMS78, C: CsEMS189, D: CsEMS201. Pop1: 1–26, Pop2: 27–46, Pop3: 47–66, Pop4: 67–86. (TIF) [file pone.0283189.s001.tif]
