# Supplementary material for: Genetic diversity of the wild ancient tea tree (Camellia taliensis) populations at different altitudes in Qianjiazhai
Source: PLoS One. 2023 Apr 18;18(4):e0283189. doi: 10.1371/journal.pone.0283189 (PMC10112783; doi:10.1371/journal.pone.0283189)
Supplement: S1 Table — (PDF) [file pone.0283189.s002.pdf]

S1 Table. 16 pairs of EST-SSR primers correlation properties.

Locus	Repeat motif	Primer sequences	Annealing temperature (℃)	Repeat times	Repeat length (bp)	Size of objective fragment (bp)
**CsEMS1**	AGA	CGATCATAGCTCATTCTCCTA	54.74	5	15	149
		AAGTCTGAGAATGGAAGGAAG	55.18			
**CsEMS4**	TTC	GAAGAACAAAAACAACAATCG	55.04	4	12	146
		ATATAACCGAAATTGCTTGGT	55.46			
**CsEMS53**	TC	CAGCAATTAAAAAGAAGCTGA	55.21	17	34	181
		GGGTTGTTGCGAGAGAAA	57.27			
**CsEMS68**	GT	ATCGGCCATATAGAGAGAGAG	55.26	6	12	152
		ACAAAGAGACCCACACACATA	55.51			
**CsEMS71**	AG	AGATTTCCCTTTCTAAGGAGAC	54.99	21	42	150
		CTTGAGTCTGTGATCTGGAAG	54.99			
**CsEMS72**	AGA	AAATCTGAAGGAGAGAAGCAC	55.33	4	12	182
		ATCTAATCGCTATCACTGTCG	54.69			
**CsEMS78**	CTG	AGGCAGACAAAGTTAAGGAGT	54.90	4	12	152
		ACCCATGTCATCATCAGACT	55.12			
**CsEMS141**	GA	TATGCCAAATAAACTCTGCAC	55.59	12	24	117
		AACAACAATGTCTTGACCAAC	55.04			

**CsEMS143**	TATGG	AGAGCTTAGCCAAGAAAAGAT	54.43	3	15	161
		CCGAGGTACCATCAATACATA	55.08			
**CsEMS146**	ATGT	GTATGGGTTTGGGTTTTTATT	54.77	3	12	162
		ATACAATTCAACCCTCCTTTC	54.84			
**CsEMS155**	TGAA	AGAAGAAGAATGGTGGCTTT	55.61	3	12	153
		CAACTTAGCATTTGAAGATGAA	54.91			
**CsEMS159**	AGA	GGGGCTGTTACTTTATTGTTC	55.50	4	12	155
		CAAGCAAGTTGGACATTTTC	55.88			
**CsEMS183**	GAG	AGAGAGCGAGGACTGAGTAAT	54.95	4	12	144
		AAAGAAGCAGAAGAGGAGTGT	55.00			
**CsEMS189**	GCG	ACATTCAAGGATGAGCAATC	55.10	5	15	151
		CACCTCCACCACCACTAC	54.40			
**CsEMS194**	AAC	CTCATCACCATCATCATCATC	56.16	6	18	133
		AACCATCATCCTTCTTGAAAC	55.71			
**CsEMS201**	AGAGA	TTTTGGTTCTTAGCTTTTGTG	54.98	3	15	112
		GCGAAAAGAATATGTTCAATG	55.09			

2
